# Tertiary center referral delay of patients with dementia in Southern Brazil: associated factors and potential solutions

**DOI:** 10.1590/1980-57642021dn15-020008

**Published:** 2021

**Authors:** Brunna de Bem Jaeger, Milena Lemos Oliveira, Raphael Machado Castilhos, Márcia Lorena Fagundes Chaves

**Affiliations:** 1 Dementia Clinic, Neurology Service, Hospital de Clínicas de Porto Alegre ‒ Porto Alegre, RS, Brazil.; 2 Post-graduate Program in Medical Sciences, Faculty of Medicine, Universidade Federal do Rio Grande do Sul ‒ Porto Alegre, RS, Brazil.; 3 Faculty of Medicine, Universidade Federal do Rio Grande do Sul ‒ Porto Alegre, RS, Brazil.; 4 Department of Internal Medicine, Faculty of Medicine, Universidade Federal do Rio Grande do Sul ‒ Porto Alegre, RS, Brazil.

**Keywords:** dementia, referral and consultation, delayed diagnosis, general practitioners, educational activities, information dissemination, demência, encaminhamento e consulta, diagnóstico tardio, clínicos gerais, atividades educativas, disseminação do conhecimento

## Abstract

Early dementia diagnosis has many benefits and is a priority. In Brazil, most cases are diagnosed by a specialist.

**Objective:**

We aimed to study the average time from disease onset to specialist assessment and related factors; we also propose potential strategies to deal with this delay.

**Methods:**

This was a cross-sectional database study in 245 patients with dementia from an outpatient clinic in a tertiary university hospital in Southern Brazil, which only assesses individuals from the Unified Health System (SUS). The outcome was time from symptoms onset to specialist assessment, reported by the informants. Individuals were separated into two groups: less and more than 1 year to specialist assessment. Multivariable analysis was used to test the potential related factors associated with delayed specialist assessment.

**Results:**

Mean±SD of time from symptoms onset to specialist assessment was 3.3±3.3 years. In the unadjusted analysis, individuals who were assessed before 1 year were more often diagnosed with vascular dementia, had more sudden and subacute onset, neuropsychiatric symptoms at presentation, rapid progression, and alcohol and antipsychotics use (p<0.05). In multivariate analysis, the effects of personality changes and onset presentation persisted, even when controlling for other variables.

**Conclusion:**

We found a long time from disease onset to specialist assessment, and those with personality changes and faster presentation were referred earlier. Improving the diagnostic capability of general practitioners, mass educational campaigns and transmission of knowledge by experts are some potential strategies to deal with delay of dementia diagnosis.

## RESUMO.

O diagnóstico precoce de demência tem muitos benefícios e deve ser uma prioridade. No Brasil, ele é feito por especialistas na maioria dos casos.

**Objetivo:**

O objetivo deste estudo foi avaliar o tempo médio entre o início da doença até a avaliação com especialista e seus possíveis fatores relacionados; também propomos estratégias potenciais para lidar com esse atraso.

**Métodos:**

Trata-se de um estudo transversal de base de dados com 245 pacientes com demência atendidos em ambulatório de um hospital universitário do sul do Brasil, que avalia indivíduos provenientes do Sistema Único de Saúde (SUS). O desfecho principal foi o tempo entre o início dos sintomas até a avaliação com o especialista, relatados pelos informantes. Os indivíduos foram separados em dois grupos: tempo até a consulta com o especialista menor e maior que 1 ano. A análise multivariável foi usada para testar os possíveis fatores relacionados à avaliação tardia pelo especialista.

**Resultados:**

O tempo médio±DP entre o início dos sintomas e a avaliação com o especialista foi de 3,3±3,3 anos. Na análise não ajustada, os indivíduos que chegaram para avaliação antes de 1 ano do início da doença foram diagnosticados com mais frequência com demência vascular, tiveram início do quadro mais repentino e subagudo, sintomas neuropsiquiátricos na apresentação, progressão rápida, uso de álcool e antipsicóticos (p<0,05). Na análise multivariada, apenas alterações de personalidade e início rápido dos sintomas mostraram-se preditores para chegada mais precoce ao especialista, mesmo controlando possíveis confundidores.

**Conclusão:**

Encontramos um longo tempo entre o início da doença até a avaliação do especialista e indivíduos com alterações de personalidade e apresentação mais rápida foram encaminhados mais precocemente. Melhorar a capacidade diagnóstica do médico de família, campanhas educacionais em massa e transmissão de conhecimento por especialistas são algumas estratégias potenciais para lidar com o atraso do diagnóstico de demência.

## INTRODUCTION

The population aging process is ongoing worldwide and poses major challenges to health care systems, especially in low-income regions, such as Latin America. An increasing prevalence of dementia is a predictable consequence of this process.^1^ Among several challenges in dementia care, both globally^2^ and especially in Latin America,^3^ timely diagnosis is essential. Early diagnosis has many benefits, such as delaying of institutionalization, decreasing caregiver burden, and reducing the costs.^4^ ^,^ ^5^ Individuals with dementia take a long time from symptoms onset to diagnosis.

Delays in dementia diagnosis have been identified in several regions,^6^ ^,^ ^7^ ^,^ ^8^ mostly in high-income countries. A recent systematic review found no study in low-income countries assessing the frequency of undetected dementia, estimating that it can reach 93%.^9^ In Brazil, few studies have been performed with the aim to assess diagnostic delays and their possible causes. Miranda et al., in evaluating factors influencing delays in the diagnosis of Alzheimer’s disease, showed a median of 1.5 years until tertiary evaluation and diagnosis.^10^

Multiple reasons are highlighted as potential barriers to the diagnosis assessment, such as system-, patient-, caregiver-, and physician-related factors.^5^ Some variables were previously identified as predictors of early specialist assessment, such as vascular dementia and neuropsychiatric symptoms.^11^ ^,^ ^12^ On the other hand, female sex, family history, frontotemporal dementia, Alzheimer’s disease, early onset dementia and low Mini-Mental State Examination (MMSE) scores were associated with delayed diagnosis.^6^ ^,^ ^11^ ^,^ ^13^ ^,^ ^14^ ^,^ ^15^ Finally, level of education has shown conflicting results.^7^ ^,^ ^10^ ^,^ ^12^ ^,^ ^13^ ^,^ ^14^ ^,^ ^15^ ^,^ ^16^

Brazil is the largest and most populous country in Latin America and has a unified public health care service whose referral system requires patients to be evaluated by a general practitioner before being referred to a tertiary center. In Southern Brazil, the state of Rio Grande do Sul will have the highest proportion of elderly citizens in the next ten years,^17^ so the evaluation of possible diagnostic delays is important for a better understanding of the functioning of the public health care system and essential for the establishment of measures that can improve this performance.

The aim of this study was to estimate the delay of referral of patients with dementia, for any reason, to a tertiary public health care outpatient dementia clinic in Southern Brazil and to determine potentially associated factors. Additionally, we explored some potential strategies to improve this delay.

## METHODS

This was a databank-based cross-sectional study with outpatients from a dementia clinic of a tertiary care university hospital in Southern Brazil, Hospital de Clínicas de Porto Alegre (HCPA), which is part of the Porto Alegre network of the Brazilian Public Health System (SUS). In our dementia clinic, we routinely evaluate patients with suspected dementia through a structured assessment, in which we collect various sociodemographic and clinical variables. Patients who had been assessed between 2014 to 2018 and met the criteria for dementia of any cause according to the criteria of McKhann et al.^18^ were included. Those who had diagnoses other than dementia and no information about the time from the first symptom to assessment were excluded. Individuals should have attended at least one standard consultation, including a thorough interview with the patient and the informant, tests for cognitive performance and a comprehensive clinical and neurological examination, besides laboratory and neuroimaging data.

The variables below were collected in the first evaluation.

- **Time from symptoms onset to specialist assessment:** The information about disease onset at first evaluation was obtained during the first interview with the informant. Patients were then divided into two groups: less than or equal to 1 year and more than 1 year of disease duration at first evaluation. The choice for classifying the sample in that way was based on previous findings of patients often taking on average more than 1 year to receive the diagnosis.^10^ ^,^ ^7^ ^,^ ^12^ ^,^ ^19^
- **Demographic data:** Age, sex, and years of education were obtained.
- **Clinical data:** Number of cardiovascular risk factors (hypertension, ischemic cardiopathy, heart failure, dyslipidemia, diabetes), neurological disorders (stroke, epilepsy, migraine), and other comorbidities as well were recorded. Additionally, the number of medications, including antipsychotics, anticholinergics, antidepressants was recorded. Tobacco and alcohol use and traumatic brain injury were also obtained.
- **Cognitive data:** For the present analysis, the Brazilian version of the Mini-Mental State Examination (MMSE)^20^ was used. Age at symptoms onset, onset presentation (abrupt/insidious), trigger events, and family history of dementia were collected.
- **Functional status and cognitive and neuropsychiatric/behavioral symptoms:** We grouped the symptoms presented in the first evaluation as: amnesia, impaired comprehension, temporal and spatial disorientation, personality changes, difficulties at work, depression, hallucinations, word finding difficulties, behavioral changes, difficulty in household chores, repeating the same questions, and delusions. We also included the scores of the Brazilian version of the Geriatric Depression Scale^21^ in the analysis.
- **Diagnostic assessment:** Patients were classified according to different types of dementia following standard international diagnostic criteria as Alzheimer’s disease,^22^ vascular dementia,^23^ Lewy body dementia,^24^ behavioral variant of frontotemporal dementia,^25^ and mixed dementia and primary progressive aphasias.^26^ ^,^ ^27^

Continuous variables were expressed as mean±standard deviation (SD) and were analyzed with Student’s t-test or the Mann-Whitney U test, where appropriate. Categorical data were presented as frequencies and were tested with the Pearson’s chi-square test with Fisher or Yates correction when necessary. A multivariate binary logistic regression model was used to determine the significant association of medical and demographic variables with the outcome "time to assessment" (≤1 year/>1 year). All analyses were considered statistically significant at a p<0.05, and all data were analyzed using the *Statistical Package for the Social Sciences* (SPSS) software (version 16.0; SPSS, Chicago, IL, USA).

This study was approved by the Human Research Ethics Committee and signed consent was obtained from all patients or a proxy.

## RESULTS

A total of 245 patients were included in the study. As summarized in Table 1, the mean age of the participants was 72.4±10 years old, and the sample consisted predominantly of women (female sex, 56.9%). The mean level of education was 5.2±3.9 years, and more than half had less than 8 years of formal schooling. The mean±SD of the MMSE score was 15.9±6.8, and the majority were diagnosed as dementia due to Alzheimer’s disease (44.7%). Of the 245 participants, 64 (31.1%) had a family history of dementia, 36 (16.6%) had a previous traumatic brain injury, 63 (29%) and 96 (43.6%) reported alcohol use and tobacco use, respectively.

**Table 1.** Clinical characteristics of the groups according to time to specialist assessment

Characteristic	Total n=245	Time to assessment		p-value
		≤1 year n=50 (20.4%)	>1 year n=195 (79.6%)	
Demographics Age (years), mean±SD	72.4±10	71.1±10.7	71.7±10.4	0.712
Female sex, n (%)	132 (56.9)	27 (54)	105 (57.7)	0.747
Education (years), mean±SD	5.2±3.9	5.6±3.8	5.3±4.1	0.969
**Diagnosis, n (%)**				0.031
Alzheimer’s disease dementia	92 (44.7)	12 (27.3)	80 (49.4)	
Vascular dementia	47 (22.8)	13 (29.5)	34 (21)	
Other types, n (%)*	67 (32.5)	19 (43.2)	48 (29.6)	
**Medical history**				
TBI, n (%)	36 (16.6)	10 (20.4)	26 (15.5)	0.512
Alcohol use, n (%)	63 (29)	20 (40.8)	43 (25.6)	0.049
Tobacco use, n (%)	96 (43.6)	27 (55,1)	69 (40.4)	0.074
Family history, n (%)	64 (31.1)	13 (28.3)	51 (31.9)	0.720
Number of CV risk factors, mean±SD	1.2±1.1	1.5±1.2	1.2±1.1	0.410
Any neurological comorbidity, n (%)	0.1±0.4	0.21±0.4	0.1±0.4	0.885
Psychiatric comorbidity, n (%)	81 (37.7)	22 (44.9)	59 (35.5)	0.245
MMSE, mean±SD	15.9±6.8	20±6.7	17.8±7.3	0.890
GDS, mean±SD	5.37±3.6	6.6±4.2	5.9±4	0.247
Age at onset (years), mean±SD	68.9±10.9	67.2±10.8	66.5±11.5	0.962
Insidious onset, n (%)	151 (70.9)	18 (37.5)	133 (80.6)	**<0.001**
Personality changes, n (%)	28 (13.6)	13 (29.5)	15 (9,3)	**0.001**
Behavior changes, n (%)	44 (21.4)	15 (34.1)	29 (17.9)	**0.024**
Visual hallucinations, n (%)	20 (9.7)	10 (22.7)	10 (6.2)	**0.003**
Slow progression, n (%)	132 (64.1)	17 (38.6)	115 (71)	**<0.001**
**Medication, n (%)**				
AChE	31 (12.6)	4 (8)	27 (13.8)	0.287
Benzodiazepines	25 (10.2)	8 (16)	17 (8.7)	0.186
Antipsychotics	58 (23.7)	19 (38)	39 (20)	**0.010**
Antidepressants	79 (32.2)	19 (38)	60 (30.8)	0.397

SD: standard deviation; TBI: traumatic brain injury; CV: cardiovascular; MMSE: Mini-Mental State Examination; GDS: Geriatric Depression Scale; AChE: anticholinesterase medications. *Lewy body dementia, behavioral variant of frontotemporal dementia, mixed dementia, primary progressive aphasias, Parkinson’s disease dementia, not specified.

The mean time from symptoms onset to specialist assessment was 3.3±3.3 years, and most patients were evaluated 1 year after disease onset (79.6%) (Table 1). In the univariate analysis, the following factors were significantly different between groups: alcohol use, onset presentation, symptoms at presentation (personality changes, behavior changes, visual hallucinations), disease progression, and antipsychotic use (Table 1). No significant difference was found for level of education, age at disease onset and cognitive impairment (MMSE scores). In the multivariate logistic regression analysis, the variables personality changes (OR=3.24; 95%CI 1.10-9.51) and onset presentation (OR=6.19; 95%CI 2.62-14.66) were kept in the final model, after controlling for other significant characteristics (Table 2).

**Table 2.** Multivariable-adjusted predictors of time to the symptom onset to specialist assessment.

	OR	95%CI	p-value
Personality changes	3.24	1.10-9.51	0.032
Behavior changes	0.91	0.34-2.43	0.857
Visual hallucinations	2.37	0.69-8.17	0.170
Sum of comorbidities	0.93	0.71-1.21	0.601
Antipsychotics	1.30	0.55-3.10	0.544
Alcohol use	1.38	0.58-3.23	0.459
Dementia type	1.45	0.56-3.76	0.436
Onset presentation	6.19	2.62-14.66	0.001

OR: *Odds Ratio*; 95%CI: 95% confidence interval.

## DISCUSSION

This study investigated the time between symptoms onset to specialist evaluation among dementia outpatients from a public tertiary care university hospital in Porto Alegre, Southern Brazil. The mean time elapsed from disease onset to specialist assessment was 3.3 year, with most of the sample (79.6%) evaluated after at least 1 year from the beginning of the clinical presentation. Many studies have reported a gap between early diagnosis and assessment,^4^ reinforcing the global dimension of this challenge. In other Brazilian studies^10^ ^,^ ^13^ this gap ranged from 1.8 to 4.1 years. Similarly, international studies have found a large variation (13.8 to 60 months).^7^ ^,^ ^12^ ^,^ ^15^ ^,^ ^19^ ^,^ ^28^

When analyzing related factors, the variables significantly associated with time to assessment were type of dementia, alcohol use, onset presentation, symptoms at presentation, disease progression and antipsychotic use. The time for assessment was longer (more than 1 year) among patients with dementia due to Alzheimer’s disease, for those with insidious presentation and slow progression of the disease. Extensive delay for diagnosis among Alzheimer’s disease patients has been reported, as well as related factors such as the lack of knowledge in differentiating memory loss in normal aging from that caused by Alzheimer’s disease and delay in seeking medical help for multiple reasons.^29^ Although controversial but present in other reports,^7^ ^,^ ^12^ ^,^ ^16^ we found no association between level of education and time to assessment. In our study, after the multivariable-adjusted analysis, only personality changes and onset presentation remained significantly associated with the outcome time of assessment. Probably caregivers/family see personality changes and neuropsychiatric symptoms stronger reasons to seek medical attention than memory or other cognitive complaints/symptoms.^11^ ^,^ ^29^

Multiple reasons can be highlighted as potential explanations for the delayed referral/diagnosis of dementia patients. Table 3 depict these reasons and propose strategies to deal with each one. Although we did not verify it directly in our study, the bureaucracy of Brazilian’s public health care referral system can lead to an excessive delay for a specialist evaluation.^30^ In the Brazilian Unified Health System (SUS), the level of care follows a hierarchical referencing model; i.e., a general practitioner (GP) can refer the patient for specialized evaluation when necessary (tertiary level). However, this hierarchical system ends up being a bottleneck in patient flow leading to the long wait for the specialist. A survey conducted in 2006 in Porto Alegre/RS showed 4 years of waiting time for neurologist assessment.^31^ Therefore, improving the capability of GPs to make the correct diagnosis and to manage neuropsychiatric manifestations could increase the absorption of this demand and shorten the time until diagnosis.^32^

**Table 3.** Reasons for delayed diagnosis of dementia and suggested strategies for improvement in Brazil.

Reason for diagnosis delay	Possible action strategies
Hierarchical referral model bureaucracy	Improve GPs’ capabilities in dementia diagnosis and treatment Increase availability of tests used to exclude potentially treatable causes of dementia in primary health care Select patients who will need evaluation in a tertiary center decrease the waiting time for tertiary center evaluation for more complex patients
Insufficient knowledge about dementia by GP	Improve GPs’ capabilities in dementia diagnosis and treatment Improve dementia education in medical curricula Knowledge transmission by experts
Insufficient knowledge about dementia by lay population	Educational campaigns on dementia, specially differentiating from normal aging Knowledge transmission by experts
Dementia stigma	Educational campaigns on dementia Knowledge transmission by experts

GP: general practitioner.

Another possible cause for the delay in dementia diagnosis is the lack of awareness of the non-benign, aging-related origin of the cognitive decline in the elderly. This is a common concept embraced by the lay population (and even among health care professionals) that cognitive complaints and minor functional decline are a manifestation of normal aging. The expected result of this misconception is a delay in seeking care.^5^ ^,^ ^33^ Indeed, in our analysis, neuropsychiatric manifestations and faster clinical courses were associated with shorter time to specialist evaluation. This could mean that mild cognitive and functional decline do not attract family attention and are not seen as a disease. In line with this hypothesis, in our study, patients with shorter disease times may simply represent memory bias, possibly meaning that the onset of disease claimed by family/caregiver would actually mean clinical worsening. Increasing knowledge about dementia is an obvious solution to deal with these misconceptions. Mass education campaigns informing about dementia manifestations and especially the differences from normal aging could increase the frequency of earlier diagnosis. Knowledge transmission about dementia by experts could also be an effective way to overcome barriers to a faster and more effective diagnosis and treatment of patients.^34^

The main limitation of this study is the recall bias, since time of symptoms onset is dependent on the participant/caregiver recollection of events or experiences that happened in the past. This was a databank-based cross-sectional study, which cannot allow the establishment of a cause-effect relation. Furthermore, since data were collected from medical records, it might have caused loss of information on variables of interest, such as socio-economic status, rural versus urban residence, number of physicians seen before the specialist evaluation and caregiver education.

In conclusion, this study from an outpatient clinic in Southern Brazil found a long time - mean of 3.3 years - from the onset of dementia symptoms to the specialist assessment. Those individuals who showed personality changes and a faster presentation were referred earlier. Since the burden of dementia is increasing mainly in low- and middle-income countries, there is a need to improve educational programs for the general public and physicians, optimize referral systems and reduce barriers to assess specialized centers. Moreover, the stigma of dementia is still a major challenge as it postpones open discussion about cognitive problems.^35^
